# Impact of advanced lung cancer inflammation index on all-cause mortality among patients with heart failure: a systematic review and meta-analysis with reconstructed time-to-event data

**DOI:** 10.1186/s40959-024-00295-1

**Published:** 2025-01-30

**Authors:** Ahmed Mazen Amin, Ramy Ghaly, Hossam Elbenawi, Abdelrahman Ewis, Ubaid Khan, Khaled S. M. Elshaer, Mohamed Abuelazm, Basel Abdelazeem, Brijesh Patel, Farris K. Timimi, Islam Y. Elgendy

**Affiliations:** 1 Faculty of Medicine, Mansoura University, Mansoura, Egypt; 2 Department of Internal Medicine, University of Missouri-Kansas City, Kansas City, MO USA; 3 Department of Cardiovascular Medicine, Mayo Clinic, Rochester, MN USA; 4 Division of Cardiology, University of Maryland School of Medicine, Baltimore, USA; 5 Faculty of Medicine, Alfaisal University, Riyadh, Saudi Arabia; 6 Faculty of Medicine, Tanta University, Tanta, Egypt; 7 Department of Cardiology, West Virginia University, Morgantown, WV USA; 8 Division of Cardiovascular Medicine, Gill Heart Institute, University of Kentucky, Lexington, KY USA

**Keywords:** Advanced lung cancer inflammation index, Heart failure, Inflammation, Nutrition, Review, Analysis

## Abstract

**Background:**

Heart failure (HF) is associated with systemic inflammation and hypercatabolic syndrome, impacting body metabolism. The advanced lung cancer inflammation index (ALI) is a novel inflammatory and nutritional biomarker. We aimed to investigate the prognostic role of ALI in patients with HF.

**Methods:**

We comprehensively searched PubMed, WOS, SCOPUS, EMBASE, and CENTRAL through October 2024. We conducted a pair-wise and prognostic systematic review and meta-analysis with a reconstructed time-to-event data meta-analysis. All analyses were performed using R V. 4.3.1. This meta-analysis was registered at PROSPERO (CRD42024535227).

**Results:**

We included five studies with 2,795 patients. In the pair-wise meta-analysis, ALI ≤ 25 was significantly associated with an increased incidence of all-cause mortality compared with ALI > 25 (risk ratio [RR] 1.73, 95% confidence interval [CI] 1.36–2.21, *P* < 0.01). On the adjusted prognostic meta-analysis, higher ALI was significantly associated with a reduction in the risk of all-cause mortality (hazards ratio [HR] 0.45, 95% CI 0.35–0.58-, *P* < 0.01). The reconstructed Kaplan Meier showed that ALI > 25 was significantly associated with a 56% reduction in the risk of all-cause mortality compared with ALI ≤ 25 (HR 0.44, 95% CI 0.38–0.50, *P* < 0.000001).

**Conclusion:**

Among patients with HF, a low ALI was associated with a higher incidence of all-cause mortality rate than those with a high ALI. These findings suggest that ALI can be used for prognostic stratification and aid clinical decision-making in HF management.

**Supplementary Information:**

The online version contains supplementary material available at 10.1186/s40959-024-00295-1.

## Introduction

Heart failure (HF) represents an emerging epidemic, affecting approximately 1% to 3% of the population, with a mortality rate ranging from 15 to 30% within one year [1]. In 2019, an estimated 56.2 million people were living with HF globally [2]. Projections suggest a 46% increase in HF prevalence from 2012 to 2030, accompanied by a substantial economic burden [3].

HF is associated with systemic inflammation and hypercatabolic syndrome, impacting body metabolism due to an imbalance between nutritional intake and energy demands [4]. Malnutrition occurs frequently with HF; therefore, nutritional assessment is recommended in patients with HF [5].

In patients admitted for HF exacerbation, protein-energy malnutrition is linked with a higher incidence of cardiogenic shock, cardiac arrest, longer hospital stays, and increased hospitalization costs [6]. Additionally, hypoalbuminemia observed in patients with acute HF correlated with increased mortality during hospitalization and also independently predicted long-term mortality [7]. Yet, a rise in albumin levels during the initial hospitalization period has been linked to a lower risk of a composite outcome comprising all-cause mortality and hospitalization within one year for individuals experiencing acute HF [8].

Several prognostic nutritional indices in HF have been tested, including the geriatric nutritional risk index (GNRI), controlling nutritional status (CONUT) score, and prognostic nutritional index (PNI) [9]. The advanced lung cancer inflammation index (ALI) is a novel inflammatory and nutritional biomarker that the body mass index (BMI), serum albumin level, and neutrophil-to-lymphocyte ratio (NLR), with smaller values reflecting more systemic inflammation and malnutrition. ALI was originally designed to detect the degree of systemic inflammation and malnutrition in non-small cell lung cancer (NSCLC); NSCLC patients with low ALI had significantly poor survival. Additionally, it is associated with poor prognosis in multiple types of cancers [10–12]. Moreover, ALI was found to be inversely associated with both all-cause and cardiovascular mortality in rheumatoid arthritis patients, suggesting its potential as a prognostic marker [13]. ALI is being investigated in HF as it encapsulates markers for systemic inflammation and malnutrition, both of which play critical roles in HF.

We aimed to perform a comprehensive meta-analysis to evaluate the prognostic role of ALI among HF patients.

## Methodology

### Protocol registration

We conducted this meta-analysis by adhering to the PRISMA statement guidelines for Systematic Reviews and Meta-Analyses [14] and the Cochrane Handbook for Systematic Reviews and Meta-Analysis guidelines [15]. We registered this meta-analysis prospectively in the International Prospective Register of Systematic Reviews (PROSPERO) under ID: CRD42024535227.

### Data sources & search strategy

We comprehensively searched the literature across PubMed, Cochrane Central Register of Controlled Trials (CENTRAL), Scopus, Web of Science, and EMBASE up to March 2024, using the keywords "advanced lung cancer inflammation index" and "heart failure". We updated our PubMed search in October 2024. The details of the search strategy are outlined in Table S1.

### Eligibility criteria

Regarding the pair-wise meta-analysis model, we included any comparative studies that met our PICO. The population (P) was patients with HF, irrespective of NYHA class, etiology of HF, or ejection fraction (EF) (HFpEF or HFrEF). The intervention (I) was a low ALI group (ALI ≤ 25). The control (C) was a high ALI group (ALI > 25). The outcome (O) was all-cause mortality. In the prognostic meta-analysis model, we included any comparative studies that reported the hazard ratio (HR) between the association of ALI and all-cause mortality. In the reconstructed time-to-event model, we included any comparative studies with published Kaplan‒Meier survival curves for HF patients across ALI subgroups.

### Study selection

After searching the databases, Covidence removed duplicates. Four reviewers (H.E., U.K., A.E., K.S.M.E., and A.M.A.) independently screened the title, abstract, and full text of the relevant records in accordance with the previously stated eligibility criteria. Any conflicts were settled by discussion.

### Data extraction

Four reviewers (H.E., U.K., A.E., K.S.M.E., and A.M.A.) independently extracted data from the included studies utilizing an Excel sheet encompassing (1) a summary sheet (study design, country, total number of participants, NYHA class, main inclusion criteria, primary outcome, and follow-up duration); (2) baseline information (number of patients in each group, sex, age, BMI, ischemic etiology, systolic blood pressure, diastolic blood pressure, heart rate, left atrial diameter, left ventricular ejection fraction, laboratory data (aspartic aminotransferase, alanine aminotransferase, creatinine, hemoglobin, C-reactive protein, chloride, potassium, sodium, NLR, and albumin), and comorbidities (hypertension and diabetes) and (3) study outcomes sheet (all-cause mortality). Conflicts were discussed and resolved by consensus.

### Risk of bias

Four reviewers (H.E., U.K., A.E., K.S.M.E., and A.M.A.) assessed the risk of bias in the pair-wise meta-analysis model using the Cochrane Risk Of Bias In Non-randomized Studies—of Interventions (ROBINS-I) tool [16]. This tool has seven domains to evaluate the bias arising from the confounding variables, participants' selection process, classification of the study groups, deviations from the protocol, missing outcomes, measurement of the outcomes, and selection of the reported results.

Four reviewers (H.E., U.K., A.E., K.E.) assessed the risk of bias in the prognostic meta-analysis model using the Quality in Prognostic Studies (QUIPS) tool [17]. This tool has six domains to evaluate the bias arising from participants' selection, study attrition, prognostic factor measurement, measurement of the outcomes, study confounding, and statistical analysis. Any conflicts were resolved by consensus.

### Statistical analysis

We conducted our statistical analysis using R Statistical Software (version 4.3.1, Foundation for Statistical Computing, Vienna, Austria).

To ensure clarity, we categorized our analysis into pair-wise meta-analysis, prognostic meta-analysis, and reconstructed time-to-event analysis.

Regarding the pair-wise and prognostic meta-analysis models, we pooled the results of all-cause mortality using the risk ratio (RR) and hazard ratio (HR), respectively, both with a 95% confidence interval (CI) using the random-effects model when there was a significant heterogeneity (I^2^ > 50%) and the fixed-effects model when heterogeneity was not significant (I^2^ < 50%). To assess heterogeneity, we used the Chi-square and I-square tests, where the Chi-square test assesses the presence of heterogeneity, and the I-square test assesses its degree. We considered an alpha level below 0.1 for the Chi-square test to denote significant heterogeneity. We conducted a sensitivity analysis, in which we excluded each study once to detect the source of the heterogeneity when there was a high degree of heterogeneity, which allowed us to evaluate how each study affected the overall results.

Regarding the reconstructed time-to-event data analysis, we reconstructed individual patient data from the published Kaplan–Meier graphs of three included studies [18–20] using the curve approach [21]. We adopted the two-stage approach outlined by Liu et al. [22] using the "IPDfromKM" R package. First, we extracted raw data coordinates (time, survival probability) of each arm of the included Kaplan–Meier curves. Then, individual patient data were reconstructed based on the raw data coordinates and the number of patients at risk at reported time points. Finally, we merged the reconstructed time-to-event data of all individual studies into a single merged dataset. We used the Cox frailty regression model to calculate the HR with 95% CI for the difference between ALI < 25 and ALI > 25. We included the gamma (γ) frailty term to assess the between-studies heterogeneity, where individual studies modeled as a random effect. Then, we used the likelihood ratio test to test the significance of this γ frailty term. Additionally, we employed a robust variance estimator to accommodate violations of the assumption of homoscedasticity, which assumes equal or similar variances across different groups being compared. We tested the proportional hazard ratio assumption with the Grambsch-Therneau test, log–log survival curve, and diagnostic plots based on Schoenfeld residuals [22]. We calculated Flexible parametric survival models with B-splines to provide HRs with 95 CI% of association between ALI and all-cause mortality, allowing a time-varying effect [23]. Finally, using the R package "survRM2", we analyzed the variation in restricted mean survival times (RMSTs) over time [24].

In the pair-wise and reconstructed time-to-event data analysis, we set the cut-off value for the ALI marker at 25 by averaging the cut-off values from the three included studies. However, we pooled the association between lg ALI and all-cause mortality in the prognostic meta-analysis model.

## Results

### Search results and study selection

**Fig. 1:**
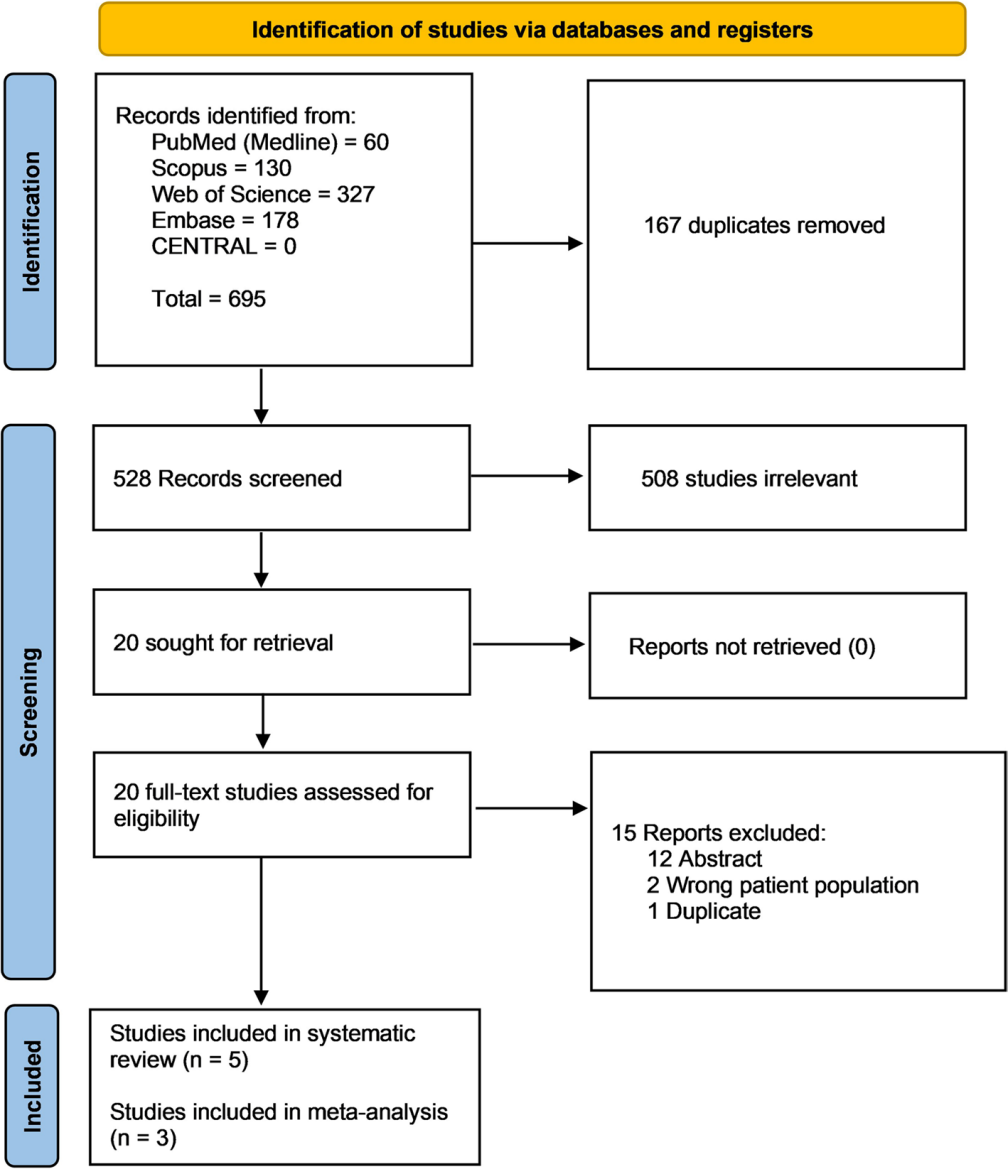
PRISMA flow chart of the screening process

After searching the following databases: PubMed, CENTRAL, Scopus, Web of Science, and EMBASE, we found 695 records. Upon 167 duplicate removals, we found 528 records eligible for title and abstract screening. After excluding 508 irrelevant studies, we found 20 records eligible for full-text screening. Finally, we included five records (Fig. 1); three studies qualified for the meta-analysis [18–20], while two studies (Kurkiewicz et al. [25] and Sun et al. [26]) were retained for the systematic review only. Their exclusion was due to the different cut-off points, which made them incompatible for pair-wise or reconstructed time-to-event data analysis. Additionally, unlike the other studies in the prognostic meta-analysis model, Sun et al. [26] and Kurkiewicz et al. [25] did not report lg ALI, making it incompatible for the prognostic meta-analysis model.

### Characteristics of included studies

We included five studies [18–20, 25, 26], with a total of 2,795 patients. There were 2,047 patients included in the pair-wise and reconstructed time-to-event data meta-analysis (1,060 patients in ALI < 25 groups and 987 in ALI > 25 groups). Meanwhile, 1,666 patients were included in the prognostic meta-analysis. More details about the characteristics of included studies and included patients are summarised in (Tables 1 and 2).

**Table 1:** Summary characteristics of the included studies

Study	Study Design	Country	Total Participants	NYHA class	Main Inclusion Criteria	Primary Outcome	Follow-up duration
**Kurkiewicz et al. 2023** [25]	Single-center, prospective cohort study	Poland	200	Classes III—IV	Patients with advanced HF ([NYHA] classes III–IV) are hospitalized for heart transplantation (HT)	All-cause mortality	One year
**Maeda et al. 2020** [19]	Single-center, retrospective cohort study	Japan	381	NA	Patients with acute decompensated heart failure (ADHF) The diagnosis of heart failure was based on the Framingham criteria	All-cause mortality and readmission because of HF	Median (IQR): 363 days (147–721) days
**Shi et al. 2023** [18]	Single-center, retrospective cohort study	China	1123	Classes III—IV	Advanced HF (NYHA classes III-IV & BNP level of > / = 500 pg/ml)	All-cause mortality	NA
**Sun et al. 2024** [26]	Multi-center, retrospective cohort study	USA	548	NA	Adult patients with HF at their initial admission to the ICU, identified using ICD-9 and ICD-10 codes. Since HF may not always be the primary diagnosis, records were included if HF appeared in any of the first five diagnosis positions	All-cause in-hospital mortality	Three months
**Yuan et al. 2022** [20]	Single-center, retrospective cohort study	China	543	Classes II-III-IV	HF patients above 65 years HF was defined according to recent guidelines as the occurrence of HF-related symptoms or signs accompanied by evidence of cardiac dysfunction, indicated by either left ventricular ejection fraction (LVEF) < 40% or elevated plasma concentration of N-terminal pro–B-type natriuretic peptide (NT-proBNP) > 125 ng/L	All-cause mortality	Five years

*NYHA* New York Heart Association, *HF* heart failure, *NA* not available

**Table 2:** Baseline characteristics of the participants

Study	Study arms	N. of patients in each group	Male, N.%	Ischemic etiology, N.%	Age (Years), Mean (SD)	BMI, Mean (SD)	SBP, Mean (SD)	DBP, Mean (SD)	HR, Mean (SD)	ALI, Mean (SD)	Left atrial diameter, mm	LVEF, Mean (SD)	Laboratory data, Mean (SD)										Comorbidities N. (%)	
													AST, U/L	ALT, U/L	Creatinie (mg/dl)	Hgb (g/dl)	CRP, (mg/dl)	Chloride (mmol/L)	Potassium (mmol/L)	Sodium (mmol/L)	NLR (mmol/L)	Albumin, g/dl	HTN	DM
**Kurkiewicz et al. 2023** [25]	**All population**	200	179 (89.5)	118 (59)	57.8 (9.3)	27.1 (4.7)	NA	NA	NA	40.4 (20.4)	52.3 (8.2)	17.3 (3.7)	26 (9)	23.3 (13.4)	1.24 (0.26)	14.18 (1.45)	NA	NA	NA	139 (3)	NA	4.3 (0.4)	114 (57)	81 (40.5)
**Maeda et al. 2020** [19]	**ALI < 23.88**	127	79 (63)	51 (40.2)	75.3 (8.9)	21.4 (4)	135.6 (35)	78.6 (17.2)	NA	15.9 (6.1)	48.3 (8.2)	43.3 (17)	23.3 (10.4)	15.3 (8.2)	1.46 (0.89)	10.8 (2.2)	0.68 (0.832)	101 (4.5)	4.3 (0.52)	138.7 (3.7)	4.1 (1.7)	3.1 (4)	90 (70.9)	49 (38.6)
	**23.88 ≤ ALI < 42.43**	127	79 (63)	37 (29.1)	75.6 (9.7)	23.8 (3.7)	133.3 (27.7)	79 (19.5)	NA	32.7 (7)	49.3 (8.2)	49.7 (21)	23.3 (10.5)	17.3 (9)	1.1 (0.43)	12 (1.7)	0.21 (0.27)	101.6 (3.7)	4.3 (0.4)	139.7 (3.7)	2.3 (0.52)	3.5 (0.4)	93 (73.3)	41 (32.2)
	**ALI ≥ 42.43**	127	67 (52.8)	31 (24.4)	72.6 (10.5)	24.4 (4.8)	138 (32.2)	80.3 (18.75)	NA	59.4 (17.9)	50.3 (8.2)	45 (18.7)	25.6 (10.5)	22 (17.24)	1.08 (0.36)	12.66 (2.5)	0.18 (0.19)	101.7 (3.7)	4.5 (0.45)	140 (3)	1.4 (0.52)	3.6 (0.45)	92 (72.4)	40 (31.5)
**Shi et al. 2023** [18]	**ALI < 24.60**	561	359 (64)	NA	69.6 (11.5)	22.26 (3.39)	121.135 (22.83)	74.91 (14.719)	87.01 (21.83)	NA	41.38 (9.55)	45.94 (18.1)	33.50 (21.9)	28.59 (20.41)	1.3 (0.53)	13.46 (2.44)	1.8 (2.2)	102.13 (5)	3.92 (0.64)	140.28 (4.84)	5.94 (3.05)	3.54 (0.47)	320 (57)	169 (30.1)
	**ALI ≥ 24.60**	562	334 (59.4)	NA	64.2 (13)	23.657 (4.14)	122.619 (22.82)	77.56 (15.24)	83.899 (20.38)	NA	43.41 (9.23)	43.30 (18.1)	28.81 (14.7)	28.44 (19.42)	1.14 (0.43)	14.18 (2.28)	0.71 (0.8)	103.66 (4.22)	3.95 (0.54)	141.78 (3.94)	2.23 (0.78)	3.79 (0.39)	300 (53.38)	146 (26)
**Sun et al. 2024** [26]	**ALI < 7.1**	181	114 (62.3)	NA	73.7 (15.7)	26.8 (5.9)	111.3 (15.5)	61.3 (10.5)	85.7 (18.1)	3.9 (2.2)	NA	NA	100.8 (131.2)	61.5 (75.1)	1.7 (1.05)	10.6 (2.4)	NA	NA	4.8 (0.8)	140.3 (4.5)	23.7 (11.4)	3.2 (0.6)	68 (37.2)	71 (38.8)
	**ALI 7.1–15.2**	183	107 (57.5)	NA	72.7 (13.7)	29.2 (6.9)	112.5 (14.3)	61.7 (10.8)	85.7 (19)	10.6 (3)	NA	NA	105 (154)	56.7 (71.7)	1.9 (1.4)	10.6 (2.9)	NA	NA	4.6 (0.8)	140 (4.5)	9.3 (3.2)	3.4 (0.5)	84 (45.2)	74 (39.8)
	**ALI ≥ 15.2**	184	108 (60.3)	NA	70.1 (15.9)	30.4 (7.3)	112.9 (16)	62.8 (9.7)	84.4 (16.5)	24.7 (10.7)	NA	NA	54.7 (53.5)	36.6 (36.5)	1.5 (1)	10.7 (2.8)	NA	NA	4.6 (0.7)	140.1 (4.3)	4.4 (1.9)	3.5 (0.5)	76 (42.5)	84 (46.9)
**Yuan et al. 2022** [20]	**ALI < 25.8**	372	188 (50.5)	205 (55.1)	77 (7.44)	22.15 (3.42)	134.75 (27.54)	75.70 (14.88)	89.40 (22.33)	13.78 (7.96)	NA	52.25 (14.1)	28.75 (14.1)	23.4 (14.88)	1.14 (0.52)	12.13 (1.94)	NA	NA	4.13(0.89)	138.65 (3.72)	6.56 (3.20)	3.76 (0.52)	243 (65.3)	115 (31)
	**ALI ≥ 25.8**	171	59 (35.5)	90 (52.6)	75.35 (8.22)	24.47 (4.34)	133 (19.43)	75.76 (14.21)	83.05 (17.19)	38.95 (13.31)	NA	55.54 (12.7)	24.35 (9.72)	21.26 (13.46)	0.94 (0.37)	12.56 (1.57)	NA	NA	4.1(.45)	140.70 (2.99)	2.6 (0.89)	4 (0.45)	114 (66.7)	4 3(25)

*N*. number, *SD* standard deviation, *BMI* body mass index, *SBP* systolic blood pressure, *DBP* diastolic blood pressure, *HR* heart rate, *ALI* advanced lung cancer inflammation index, *LVEF* left ventricular ejection fraction, *AST* aspartate aminotransferase, *ALT* alanine aminotransferase, *Hgb* haemoglobin, *CRP* C-reactive protein, *NLR* neutrophil-to-lymphocyte ratio, *HTN* hypertension, *DM* diabetes mellitus, *NA* not available

### Risk of bias

Regarding the pair-wise meta-analysis, after assessing the risk of bias by ROBINS-I, all the included studies had an overall moderate risk of bias due to bias in the confounding variables and additional bias due to missing data in three studies, as outlined in Figure S1.

Regarding the prognostic meta-analysis, after assessing the risk of bias by QUIPS, all the included studies in this model had an overall moderate risk of bias due to bias in prognostic factor measurement, as outlined in Figure S2.

### Pair-wise meta-analysis

**Fig. 2:**
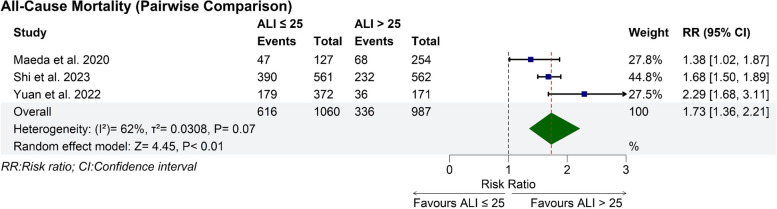
Pair-wise meta-analysis forest plot of all-cause mortality. RR: risk ratio, CI: confidence interval

This analysis included three studies with a total of 2,047 patients [18–20]. ALI ≤ 25 was significantly associated with an increased incidence of all-cause mortality compared to ALI > 25 (RR: 1.73 with 95% CI [1.36, 2.21], *P* < 0.01) (Fig. 2). Pooled studies were heterogeneous (I^2^ = 62%, *P* = 0.07). Heterogeneity was best resolved by excluding Yuan et al. (I^2^ = 29%) (Figure S3).

### Prognostic meta-analysis

**Fig. 3:**
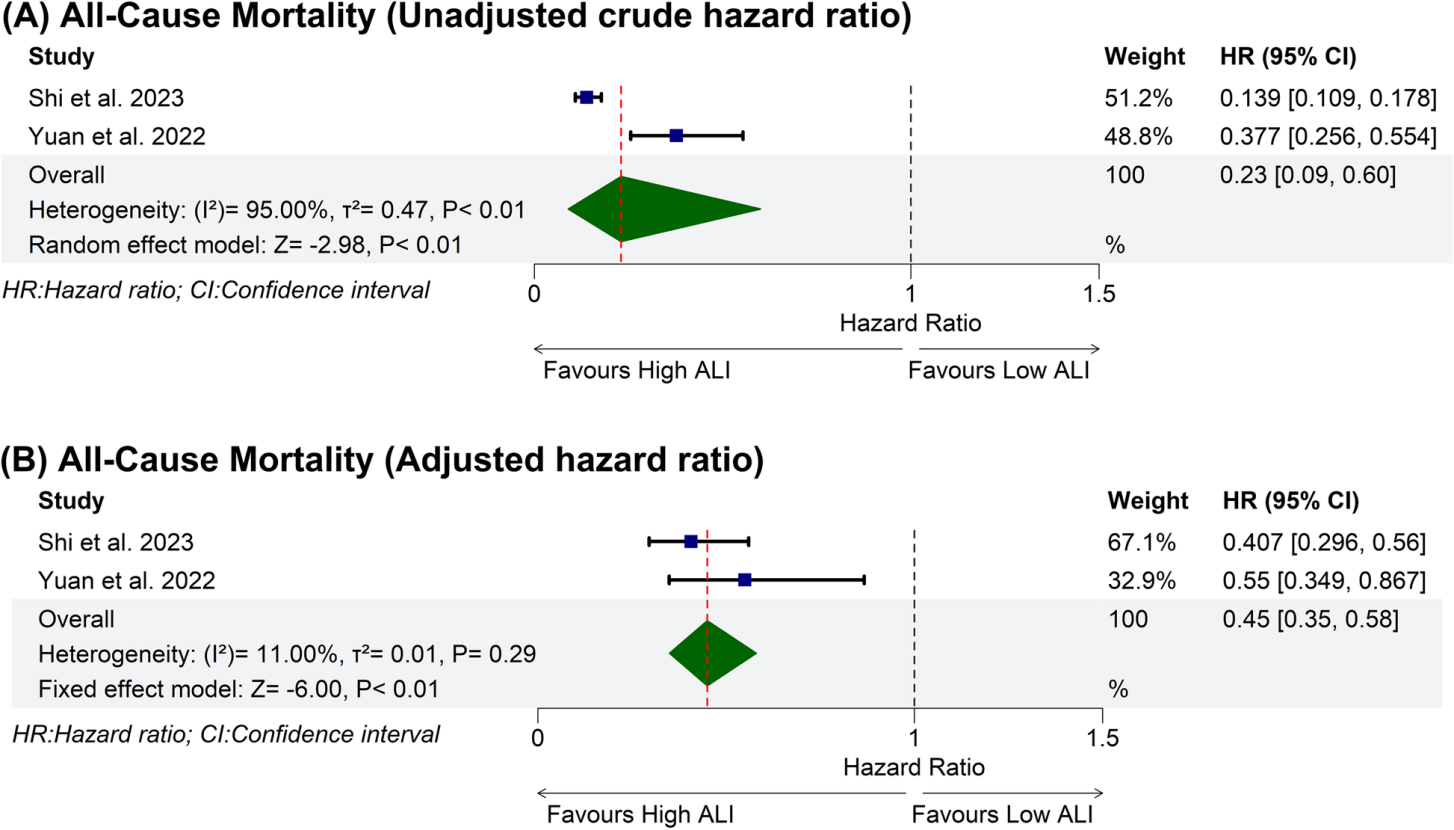
Prognostic meta-analysis forest plot of all-cause mortality. HR: hazard ratio, CI: confidence interval

This analysis included two studies with a total of 1,666 patients [18, 20]. Higher ALI was significantly associated with a 77% unadjusted reduction in the risk of all-cause mortality (HR: 0.23 with 95% CI [0.09, 0.60], *P* < 0.01). Pooled studies were heterogeneous (I^2^ = 95%, *P* < 0.01). Sensitivity analysis was not applicable (Fig. 3A). In the adjusted analysis, higher ALI was significantly associated with a 55% reduction in the risk of all-cause mortality (HR: 0.45 with 95% CI [0.35, 0.58], *P* < 0.01). Pooled studies were homogenous (I^2^ = 11%, *P* = 0.29) (Fig. 3B). The adjustment factors in each study in the adjusted analysis are outlined in Table S2.

### Reconstructed time to event data

**Fig. 4:**
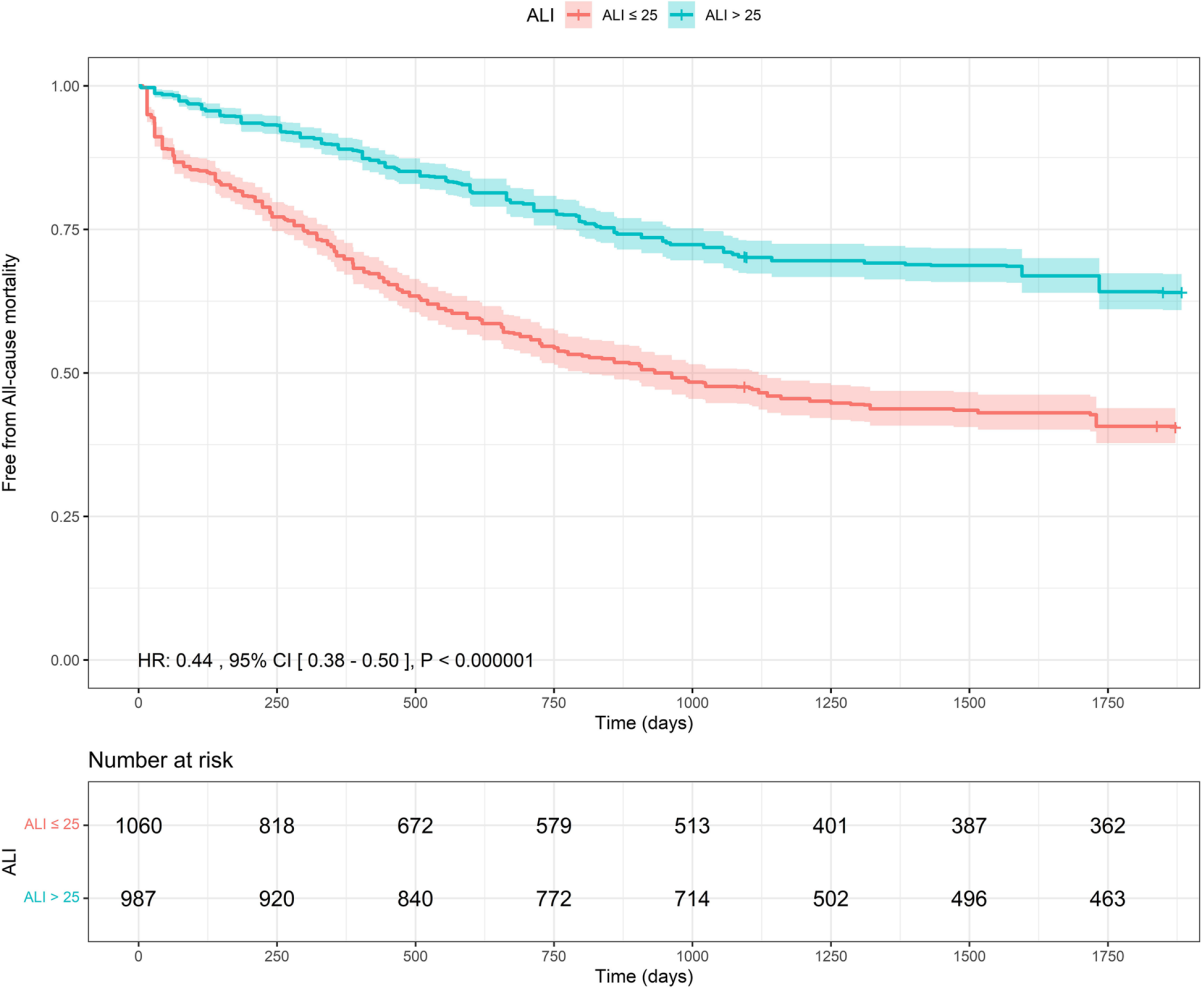
Pooled Kaplan–Meier curve showing the overall survival of ALI < 25 vs ALI > 25 heart failure patients

This analysis included three studies with a total of 2,047 patients [18–20]. During 1,750 days of follow-up, ALI > 25 was significantly associated with a 56% reduction in the risk of all-cause mortality compared to ALI < 25 (HR: 0.44 with 95% CI [0.38, 0.50], *P* < 0.000001) as shown in (Fig. 4). Additionally, there was statistically significant heterogeneity between studies (likelihood ratio test, *P* < 0.001) (Table S3).

However, upon investigating the Schoenfeld residuals plot and log–log survival curve, the proportionality of the hazard ratio over time was visually violated, and the Grambsch-Therneau test was statistically significant (*P* < 0.00001) (Figures S4 and S5).

**Fig. 5:**
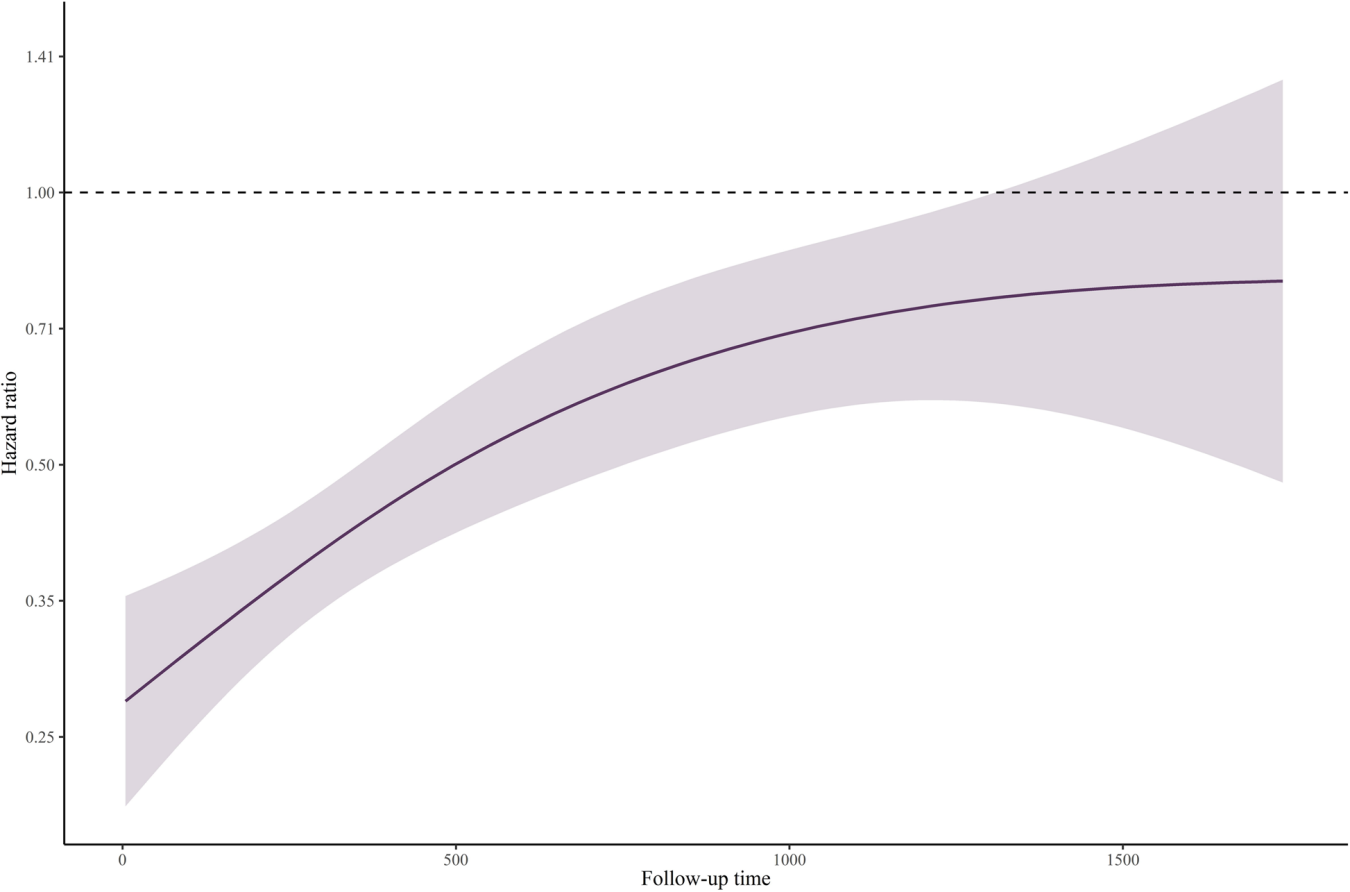
Analysis of time-varying hazard ratios for mortality based on flexible parametric survival models with B-splines

Figure 5 shows time-varying HRs for all-cause mortality based on flexible parametric survival models with B-splines, which revealed a statistically significant decrease in the risk of all-cause mortality in ALI > 25 (HR < 1) along the follow-up period.

**Fig. 6:**
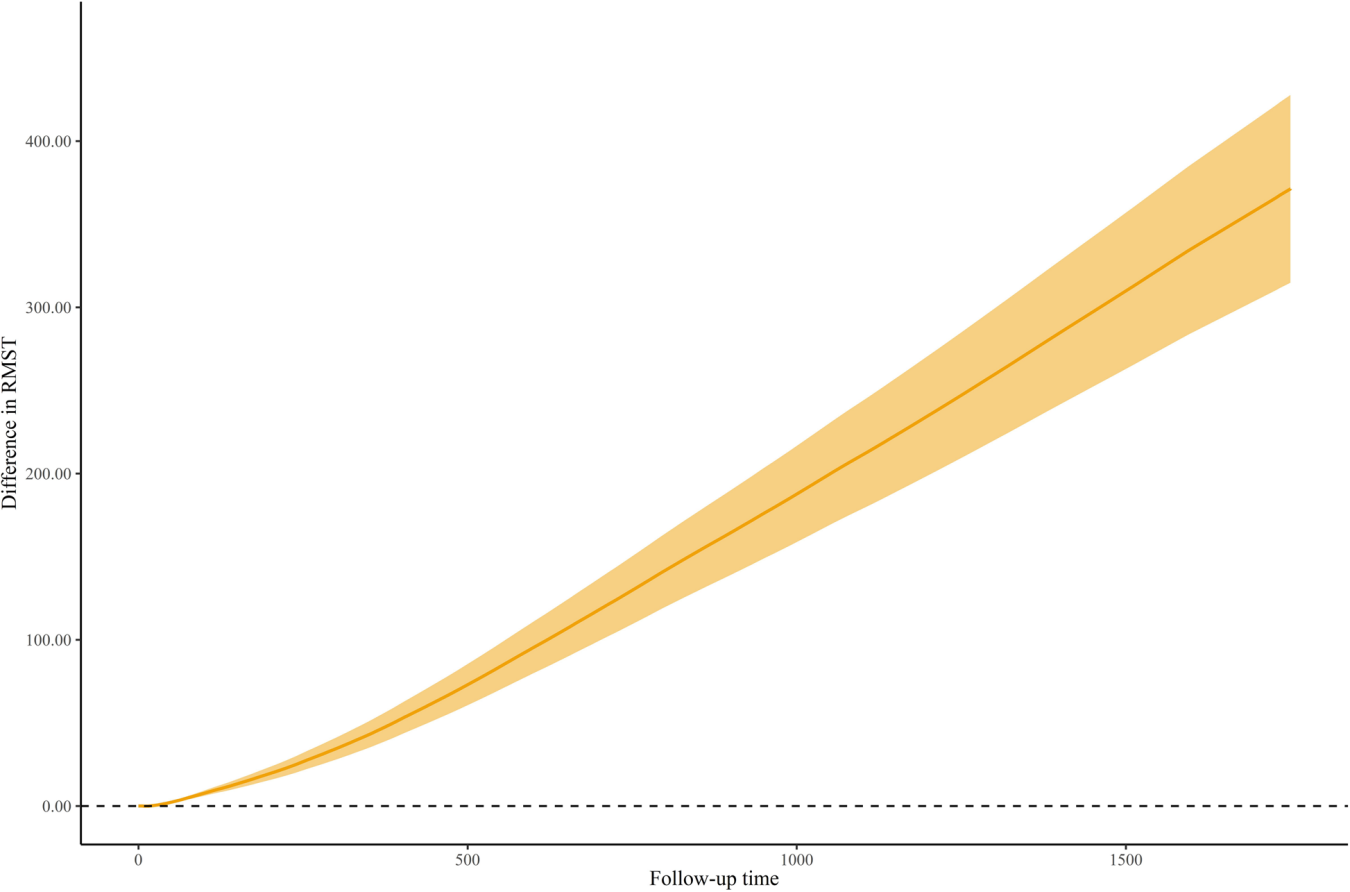
Restricted mean survival time over the entire follow-up

The difference in RMST along the follow-up period was presented in (Fig. 6) revealing that ALI > 25 is associated with a statistically significant long survival time from all-cause mortality of 399.79 days (95% CI, 338.78—460.80, *P* < 0.00001).

## Discussion

This study investigated the prognostic role of ALI among patients with HF. Our results showed that patients with low ALI were associated with a significantly higher all-cause mortality rate compared to those with high ALI. These findings suggest that ALI might be a robust marker for risk stratification and potentially guide therapeutic interventions in HF management by applying aggressive and more advanced treatment measures to higher-risk patients with lower ALI.

Several studies found that a low ALI was independently associated with higher all-cause mortality [19, 25] and HF readmission [19] in patients with HF. Sun et al. showed that ALI at admission is a significant independent predictor of both in-hospital and 90-day mortality in critically ill patients with HF [26].

Other studied prognostic indices include GNRI, CONUT, and PNI. PNI was evaluated in 1,048 HF patients over 36 months and showed a significant association with all-cause mortality (HR: 1.787, 95% CI: 1.451–2.201, *P* < 0.001) [27].

GNRI reflecting moderate to severe malnutrition risk was evaluated in 1,677 HFpEF patients over a median follow-up of 2.9 years and was significantly associated with an increased risk of all-cause mortality (HR: 1.79; 95% CI: 1.33–2.42) [28].

Yoshihisa et al. evaluated PNI, GNRI, and CONUT in 1,307 patients with HF and showed that PNI and GNRI were statistically equal and superior to CONUT in predicting mortality. In their Cox regression model, each index was an independent predictor of all-cause mortality over 1,146 days of follow-up (*P* < 0.001). The receiver operating characteristic curve demonstrated that the areas under the curve for PNI and GNRI were the same and larger than those for the CONUT score [29].

Studies investigating the prognostic significance of ALI and GNRI found that both ALI and GNRI were independent predictors of all-cause mortality [18, 20] and cardiovascular mortality [20] in HF patients.

Shi et al. compared the prognostic value of ALI and GNRI in 1,123 HF patients and showed that ALI was superior to GNRI in predicting mortality; the receiver operating characteristic curve showed that ALI had a larger area under the curve than GNRI (0.704 vs. 0.633) [18].

The above studies have demonstrated that ALI provides better prognostic value than other frequently utilized indices. In contrast to other prognostic indices, ALI strongly represents chronic systemic inflammation status, incorporating BMI, serum albumin, and NLR. However, GNRI includes serum albumin and total body weight, CONUT incorporates serum albumin, cholesterol, and TLC, and PNI consists of serum albumin and TLC [9].

We set the cut-off value for the ALI marker at 25 by averaging the cut-off values from the three included studies. Additionally, Yuan et al. showed that a value of 25.8 was 83.5% sensitive in detecting all-cause mortality [20]. Our results validate the prognostic value of ALI among HF patients. ALI showed a significant association with all-cause mortality among HF patients across three different models.

There is notable variation in ALI across different age and sex groups. Sun et al. reported that patients with lower ALI were predominantly older and male [26]. In the study by Yuan et al. [20], the median ALI value (18.73) was lower than that observed by Maeda et al. [19] (33.01), likely due to the older median age (77 vs. 75 years) and a higher proportion of female patients (54.5% vs. 40.9%). Several studies have shown an inverse relationship between age and ALI, which might be explained by the higher prevalence of malnutrition in older patients and the effects of aging on cardiovascular function, leading to an increase in oxidative stress and chronic low-grade inflammation [30].

ALI is used in cancers and systemic inflammatory diseases [10]. It is a simple and inexpensive index calculated as BMI × albumin level/NLR. BMI and albumin reflect nutritional status, while NLR reflects systemic inflammation.

There is evidence that low BMI is associated with an increased risk of mortality and hospitalization in patients with HF [31]. Hypoalbuminemia has been associated with worse outcomes in chronic diseases like HF. It leads to a reduction in colloidal osmotic pressure, which increases systemic and pulmonary fluid retention [32, 33]. Hypoalbuminemia in acute HF patients was associated with higher hospital mortality and long-term mortality [7].

NLR measures the balance between neutrophils and lymphocytes. An increase in NLR can occur from a rise in neutrophils or a drop in lymphocytes, indicating a state of systemic inflammation [34]. NLR is a component of the ALI index, which has been shown to predict mortality in different clinical situations, such as lung cancer [35].

While elevated NLR, as a marker of inflammation and immune deficiency, has been associated with poorer outcomes in HF patients [36], the ALI index offers a promising tool for predicting prognosis due to its multidimensional approach. ALI index also incorporates BMI and serum albumin in addition to NLR, providing a more comprehensive assessment of both inflammatory and nutritional status, making it particularly relevant in HF.

Inflammation can be both a cause and consequence of HF and plays a central role in disease pathogenesis and progression. Comorbidities that commonly coexist with HF, including diabetes, obesity, and chronic kidney disease, create an environment of chronic low-grade inflammation. Additionally, activation of the innate and humoral immune system, endothelial inflammation, and inflammatory mediators from the gastrointestinal tract, spleen, and adipose tissue harm the cardiac structure and function [37]. Chronic inflammation promotes monocyte infiltration into the myocardium and their differentiation into pro-inflammatory macrophages (M1) [38]. These events promote adverse left ventricle remodeling [38]

Certain trials have demonstrated that anti-inflammatory medications such as anakinra [39] could enhance coronary flow reserve, aortic distensibility, myocardial contractility, and relaxation. They also indicated a greater recovery in left ventricular ejection fraction (LVEF) in patients with acute decompensated HF, highlighting the significant role of inflammation in the progression of HF.

Additionally, in an RCT, combining a Mediterranean diet with omega-3-enriched oral nutritional supplements reduced levels of certain inflammatory cytokines in HF patients, suggesting potential benefits for heart function [40].

Therefore, ALI encapsulates systemic inflammation and nutritional status, two pivotal factors influencing HF progression. It could serve as a dual-function marker that helps identify high-risk patients who might benefit from aggressive management strategies.

## Limitations

This review should be interpreted considering the following limitations: 1) heterogeneous baseline patient characteristics. 2) predominantly male representation. 3) There is a predominantly Asian population in three out of five studies; therefore, results might not apply to other races. 3) lack of established cause-effect relationship between ALI and worsening HF. 4) in patients with an obesity phenotype of HFpEF, high BMI might elevate ALI without being associated with improved clinical outcomes. 5) BMI might be falsely elevated in patients with fluid retention, leading to a higher ALI. 6) While all studies, except for Kurkiewicz et al. [25], focused on patients with acute-on-chronic HF, data on the role of ALI in chronic heart failure is limited. 7) We could not analyze in-hospital and cardiovascular mortality outcomes because of the limited data. 8) Only Sun et al. commented on rates of coexisting cancers at baseline. Finally, while the included studies adjusted for several prognostic factors, the specific factors varied between studies.

## Implications for future research

Future-powered studies are needed to investigate the impact of ALI integration into clinical decision-making and whether ALI modification decreases mortality and hospitalizations and improves the quality of life in patients with HF. Exploring the prognostic role of ALI across the spectrum of HF ejection fraction subtypes and HF phenotypes is an essential area for exploration. Targeting the nutritional and inflammatory pathways shared by heart failure and its related conditions could be a promising treatment strategy. Additionally, prospective studies comparing ALI to other nutritional indices are needed.

## Conclusion

Among patients with HF, an ALI < 25 was associated with higher all-cause mortality in both pair-wise and reconstructed time-to-event analysis models. Similarly, a lower ALI significantly predicted higher all-cause mortality in the prognostic model. These findings suggest that ALI can be used for prognostic stratification and aid clinical decision-making in HF management. Further research is warranted to determine optimal ALI cut-offs for effective risk stratification in HF patients.

## Supplementary Information

Supplementary Material 1: Tables S1-S3. Figures S1-S5.

## Data Availability

No datasets were generated or analysed during the current study.
