# Supplementary material for: Impact of advanced lung cancer inflammation index on all-cause mortality among patients with heart failure: a systematic review and meta-analysis with reconstructed time-to-event data
Source: Cardiooncology. 2025 Jan 30;11:9. doi: 10.1186/s40959-024-00295-1 (PMC11780879; doi:10.1186/s40959-024-00295-1)
Supplement: Supplementary file 1 — Supplementary Material 1: Tables S1-S3. Figures S1-S5. [file 40959_2024_295_MOESM1_ESM.docx]

**Title.**

Impact of Advanced Lung Cancer Inflammation Index on All-Cause Mortality among Patients with Heart Failure: A Systematic Review and Meta-analysis with Reconstructed Time-to-Event Data.

**Running Title.**

Advanced lung cancer inflammation index for heart failure

**Authors.**

Ahmed Mazen Amin^1^, Ramy Ghaly^2^, Hossam Elbenawi^3^, Abdelrahman Ewis^1^_,_ Ubaid Khan^4^, Khaled S M Elshaer^5^, Mohamed Abuelazm^6^, Basel Abdelazeem^7^, Brijesh Patel^7^, Farris K. Timimi^3^, Islam Y. Elgendy^8^.

**Affiliations.**

1. Faculty of Medicine, Mansoura University, Mansoura, Egypt.
2. Department of Internal Medicine, University of Missouri-Kansas City, Kansas City, MO, USA.
3. Department of Cardiovascular Medicine, Mayo Clinic, Rochester, Minnesota, USA.
4. Division of Cardiology, University of Maryland, School of Medicine, Baltimore, USA.
5. Faculty of Medicine, Alfaisal University, Riyadh, Saudi Arabia.
6. Faculty of Medicine, Tanta University, Tanta, Egypt.
7. Department of Cardiology, West Virginia University, Morgantown, West Virginia, USA.
8. Division of Cardiovascular Medicine, Gill Heart Institute, University of Kentucky, Lexington, KY, USA.

**Contents:**

**Figures.**Figure S1: Quality assessment of the risk of bias in the included trials (ROBINS-I tool). The upper panel presents a schematic representation of risks (low = green, unclear = yellow, and high = red) for specific types of biases of each study in the review. The lower panel presents risks (low = green, unclear = yellow, and high = red) for the subtypes of biases of the combination of studies included in this review.

Figure S2: Quality assessment of the risk of bias in the included trials (QUIPS tool). The upper panel presents a schematic representation of risks (low = green, unclear = yellow, and high = red) for specific types of biases of each study in the review. The lower panel presents risks (low = green, unclear = yellow, and high = red) for the subtypes of biases of the combination of studies included in this review.

Figure S3: Sensitivity analysis of all-cause mortality (pairwise meta-analysis).

Figure S4: Grambsch-Therneau test and diagnostic plots based on Schoenfeld residuals.

Figure S5: log-log survival curve.

**Tables.**Table S1: Search strategy.

Table S2: Adjustment factors for each study's prognostic meta-analysis model.

Table S3: between-study heterogeneity assessment by frailty model.


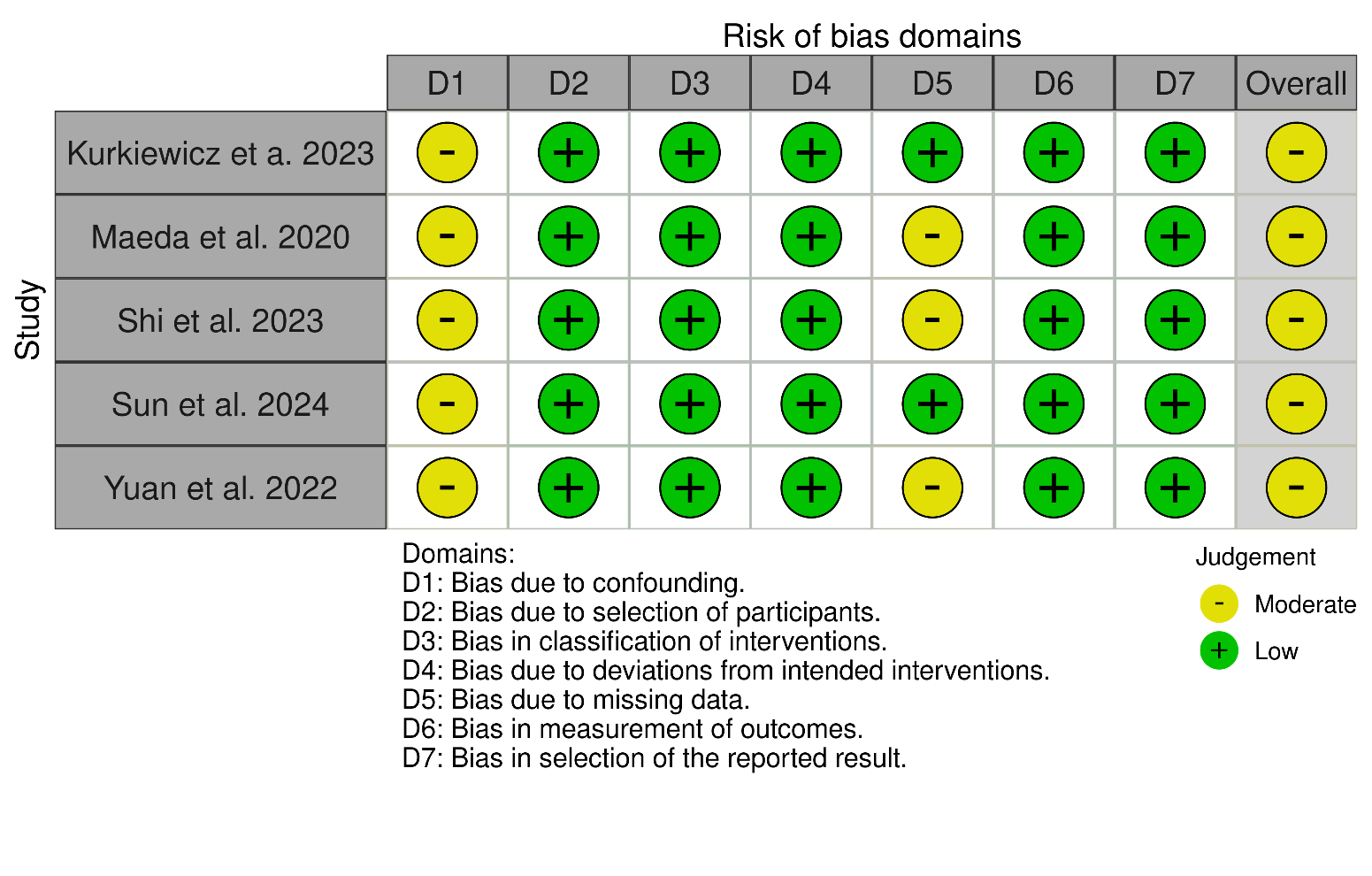


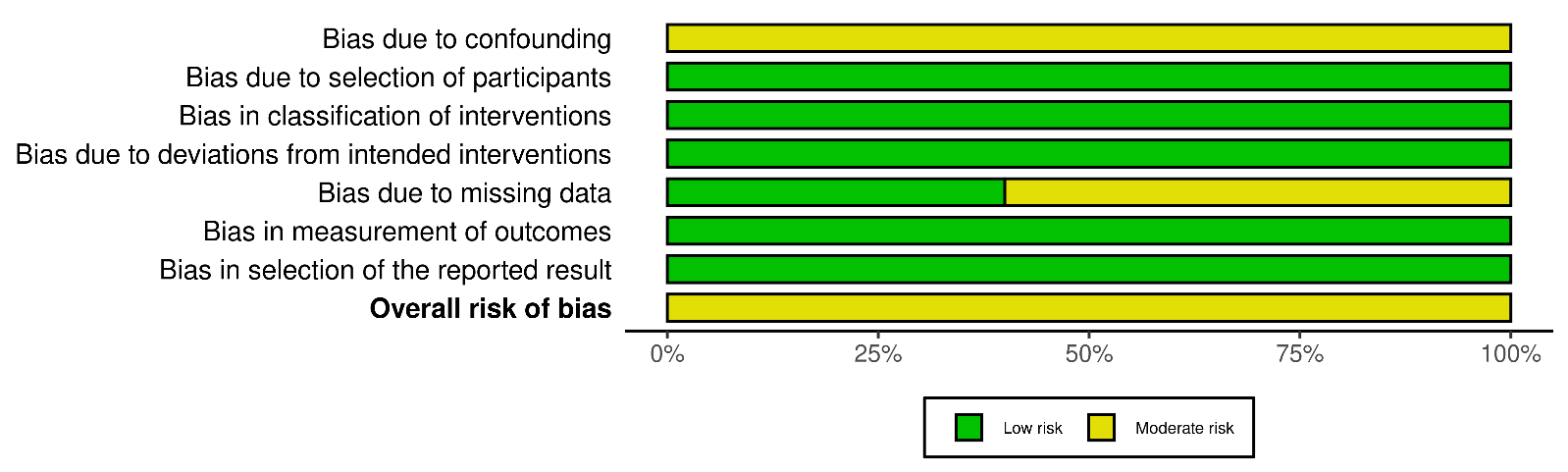


**Figure S1: Quality assessment of risk of bias in the included trials (ROBINS-I tool). The upper panel presents a schematic representation of risks (low = green, unclear = yellow, and high = red) for specific types of biases of each study in the review. The lower panel presents risks (low = green, unclear = yellow, and high = red) for the subtypes of biases of the combination of studies included in this review.**


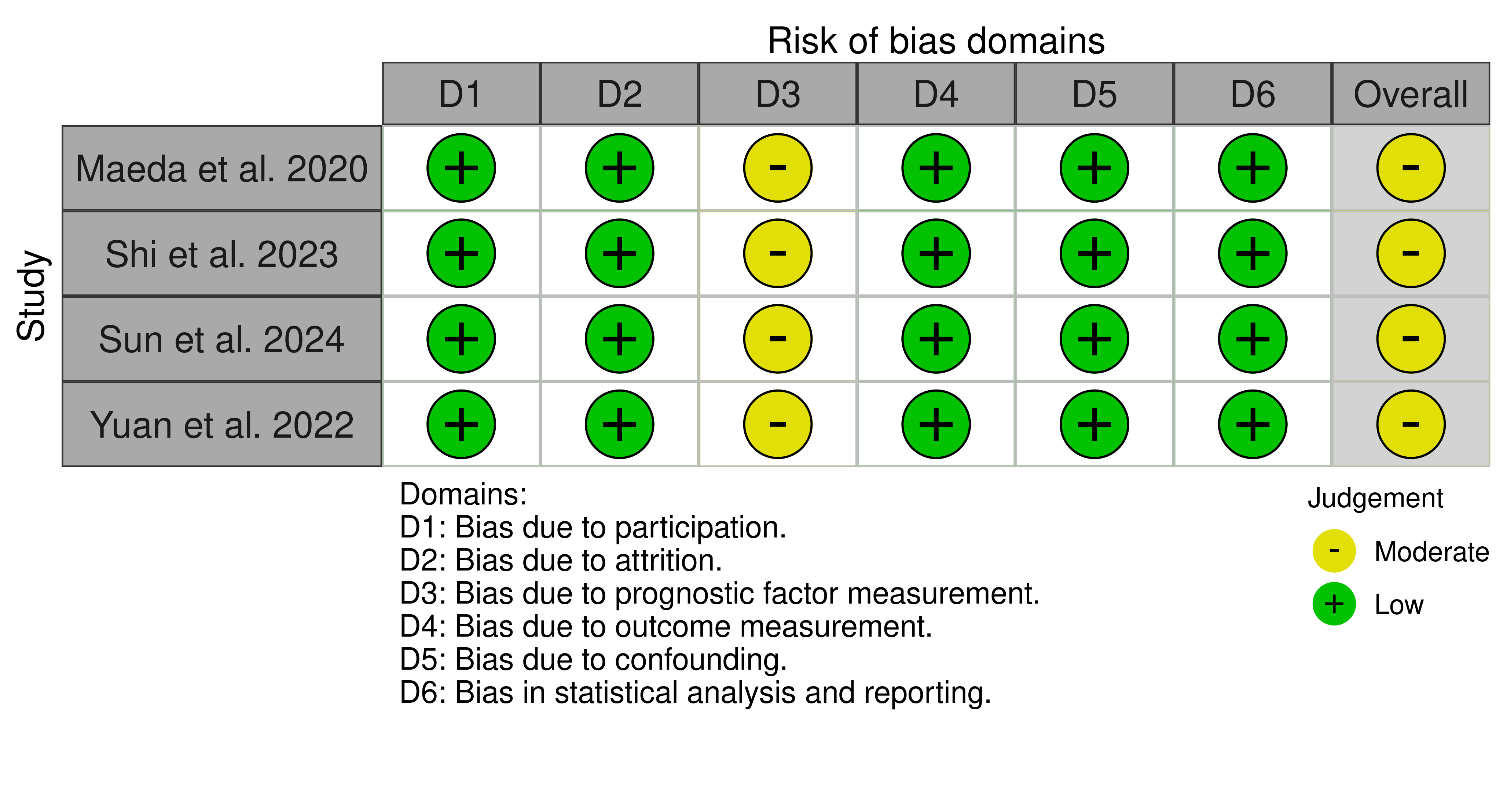

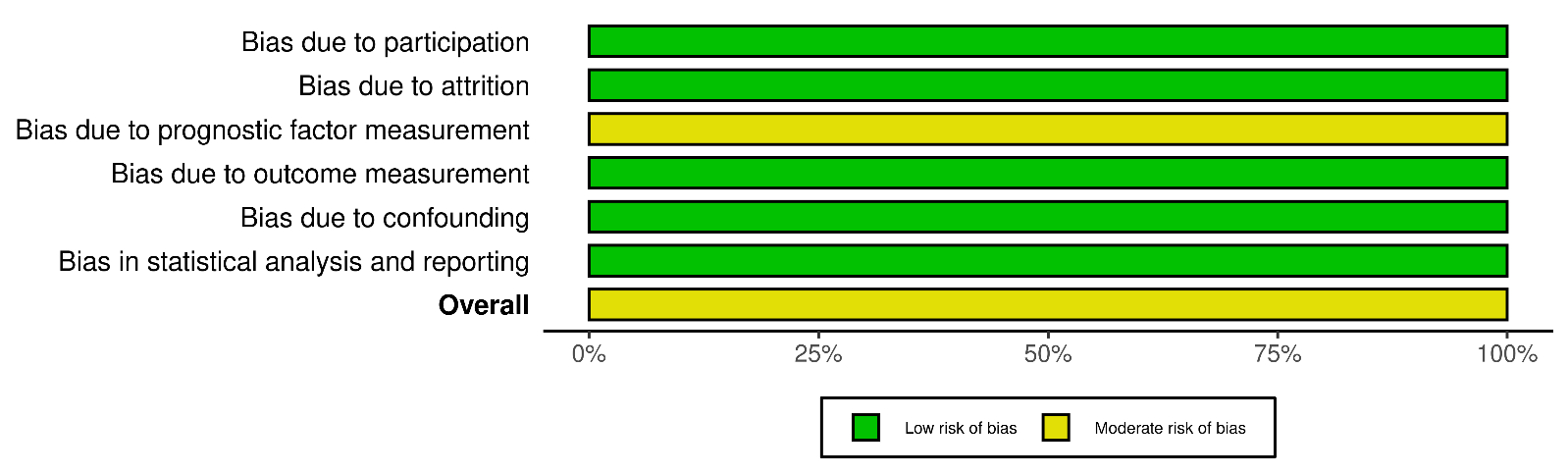


**Figure S2: Quality assessment of risk of bias in the included trials (QUIPS tool). The upper panel presents a schematic representation of risks (low = green, unclear = yellow, and high = red) for specific types of biases of each study in the review. The lower panel presents risks (low = green, unclear = yellow, and high = red) for the subtypes of biases of the combination of studies included in this review.**


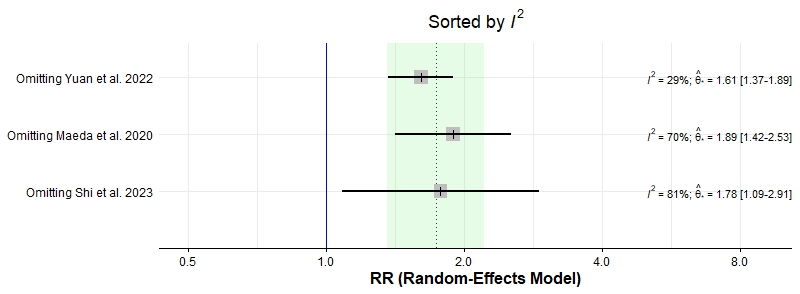


**Figure S3: Sensitivty analysis of all-cause mortality (pairwise meta-analysis).**


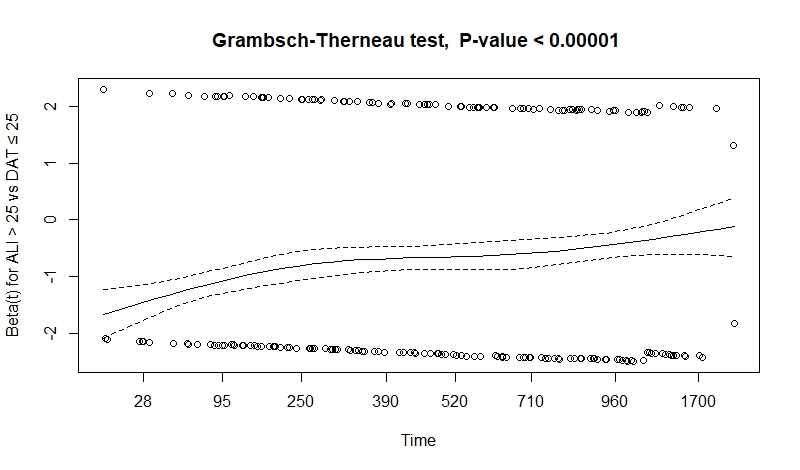


**Figure S4: Grambsch-Therneau test and diagnostic plots based on Schoenfeld residuals.**


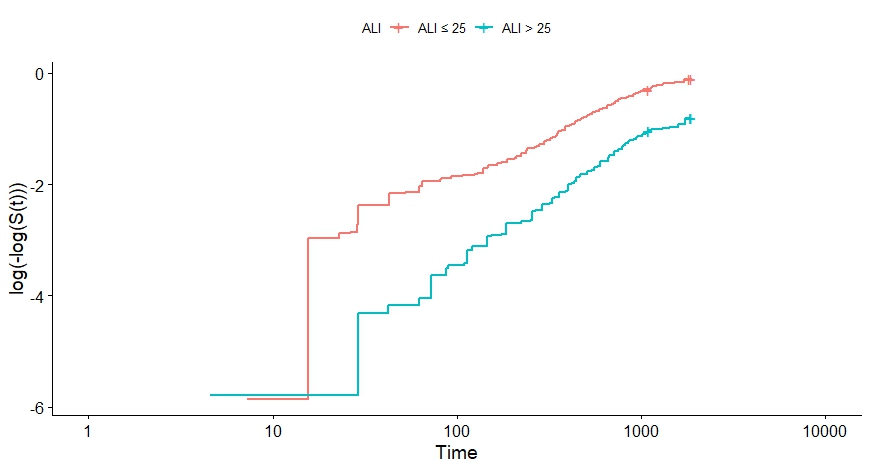


**Figure S5: Log-log survival curve.**

| Database | Search Terms | Search Field | Search Results |
| --- | --- | --- | --- |
| Pubmed | ("advanced lung cancer inflammation index" OR ALI[Title/Abstract]) AND ("heart failure" OR "cardiac failure" OR "heart decompensation" OR HFrEF OR HFpEF) | All Field | 59 |
| Cochrane | ("advanced lung cancer inflammation index") AND ("heart failure" OR "cardiac failure" OR "heart decompensation" OR HFrEF OR HFpEF) | All Field | 0 |
| WOS | 1: ((ALL=(advanced lung cancer inflammation index)) OR TI=(ALI)) OR AB=(ALI) Results: 18709  2: ((((ALL=(heart failure)) OR ALL=(cardiac failure)) OR ALL=(heart decompensation)) OR ALL=(HFrEF )) OR ALL=(HFpEF) Results: 442070  3: #2 AND #1 Results: 327 | All Field | 327 |
| SCOPUS | ("advanced lung cancer inflammation index" OR ALI) AND ("heart failure" OR "cardiac failure" OR "heart decompensation" OR HFrEF OR HFpEF) | Title, Abstract, Keywords | 130 |
| EMBASE | #3. #1 AND #2 178  #2. 'heart failure':ti,ab,kw OR 'cardiac 404,368  decompensation':ti,ab,kw OR 'heart failure with  reduced ejection fraction':ti,ab,kw OR 'heart  failure with preserved ejection  fraction':ti,ab,kw  #1. 'advanced lung cancer inflammation 18,976  index':ti,ab,kw OR ali:ti,ab,kw | All Field | 178 |

**Table S1: Search Strategy.**

| Study | Adjustment factors |
| --- | --- |
| Shi et al. 2023 | Age, NYHA class (reference: class IV), diastolic blood pressure, CRP, AST, Uric acid, Chlorine, Lg BNP, and GNRI. |
| Yuan et al. 2022 | Diastolic blood pressure, AST, Lg NT-proBNP. |

***Table S2: Adjustment factors for each study's prognostic meta-analysis model.***

CRP: C-reactive protein, AST: aspartate aminotransferase, BNP: brain natriuretic peptide, GNRI: geriatric nutritional risk index.

| Outcome | Chi-square | p-value |
| --- | --- | --- |
| All-cause mortality | 82.4 | <0.001 |

**Table S3: between-study heterogeneity assessment by frailty model.**
